# Behavioral responses of honey bees (*Apis mellifera*) to natural and synthetic xenobiotics in food

**DOI:** 10.1038/s41598-017-15066-5

**Published:** 2017-11-21

**Authors:** Ling-Hsiu Liao, Wen-Yen Wu, May R. Berenbaum

**Affiliations:** Department of Entomology, University of Illinois at Urbana-Champaign, Urbana, IL 61801-3795 USA

## Abstract

While the natural foods of the western honey bee (*Apis mellifera*) contain diverse phytochemicals, in contemporary agroecosystems honey bees also encounter pesticides as floral tissue contaminants. Whereas some ubiquitous phytochemicals in bee foods up-regulate detoxification and immunity genes, thereby benefiting nestmates, many agrochemical pesticides adversely affect bee health even at sublethal levels. How honey bees assess xenobiotic risk to nestmates as they forage is poorly understood. Accordingly, we tested nine phytochemicals ubiquitous in nectar, pollen, or propolis, as well as five synthetic xenobiotics that frequently contaminate hives—two herbicides (atrazine and glyphosate) and three fungicides (boscalid, chlorothalonil, and prochloraz). In semi-field free-flight experiments, bees were offered a choice between paired sugar water feeders amended with either a xenobiotic or solvent only (control). Among the phytochemicals, foragers consistently preferred quercetin at all five concentrations tested, as evidenced by both visitation frequency and consumption rates. This preference may reflect the long evolutionary association between honey bees and floral tissues. Of pesticides eliciting a response, bees displayed a preference at specific concentrations for glyphosate and chlorothalonil. This paradoxical preference may account for the frequency with which these pesticides occur as hive contaminants and suggests that they present a greater risk factor for honey bee health than previously suspected.

## Introduction

The western honey bee (*Apis mellifera*) is a eusocial species whose foragers collect food to meet hive requirements and adjust their food-gathering behavior according to these collective needs. Foragers are the first members of the colony to encounter and evaluate potential food resources and to make decisions about whether to bring them back to the hive. Thus, the discriminative abilities and behavioral preferences of foragers have tremendous impacts on the nutrition and health of the entire colony. Relative to other insect genomes, the *A. mellifera* genome has a strikingly reduced inventory of gustatory receptors, with the 10 gustatory receptor genes (Grs) representing only 13–15% of those present in other insect genomes^1^. Despite this reduced inventory, honey bees are demonstrably able to differentiate among select natural and synthetic chemicals^2–4^.

Phytochemicals in nectar and pollen can both attract pollinators and repel inappropriate floral visitors^5^, including honey bees. Quinine, an alkaloid from *Cinchona* species, is among the best-known phytochemical repellents for honey bees^2^. As well, some phenolic compounds in sugar water or nectar can enhance honey bee visitation^6–8^, whereas others can, depending on concentration, deter feeding^6,8–10^. Liu *et al*.^10^ speculated that foragers can estimate the concentration of phenolics in pollen and change their foraging dynamics accordingly. These findings suggest that bees have the ability to evaluate food quality and use phytochemicals as cues to make foraging decisions, but whether they rely on phytochemicals that enhance colony health as phagostimulants or whether social cues from nestmates influence nectar-gathering behavior has not yet been systematically assessed.

In addition to its nutrient content, honey, the product of processed nectar, provides phytochemicals that can promote colony health in several ways. Gherman *et al*.^11^, *e.g.*, demonstrated that nurse bees infected with *Nosema* preferentially consume sunflower honey, which has the highest antimicrobial activity among the four types of honey offered as choices. Additionally, caffeine, an alkaloid found in the nectar of species in the Rutaceae and Rubiaceae, among others, can enhance memory in honey bees^3^. Moreover, phytochemicals in nectar, honey, pollen, or propolis can confer other health benefits. The phenolic acid *p-*coumaric acid, a constituent of many honeys, upregulates both detoxification genes and immunity genes in larval and adult honey bees; bees consuming *p*-coumaric acid in sugar diet were capable of 60% higher rates of metabolism of the organophosphate acaricide coumaphos than bees consuming sugar diet alone^12,13^. Quercetin, a flavonol found in many honeys, essentially all pollen, and in propolis in many parts of the world, also upregulates at least 12 genes encoding cytochrome P450 monooxygenases, including CYP9Q1, CYP9Q2, and CYP9Q3, which detoxify both tau-fluvalinate and coumaphos^14^ and enhances longevity of workers exposed to the pyrethroid insecticide β-cyfluthrin^15^. Additionally, a sucrose diet containing both quercetin and *p*-coumaric acid enhanced the longevity of bees exposed to bifenthrin^15^.

In contrast with at least some phytochemicals, exposure to pesticides rarely if ever is beneficial to bees; rather, pesticide ingestion is associated with a wide array of negative effects^16^. Pesticides detected in honey and beebread in North American hives include insecticides, acaricides, fungicides, and herbicides^17,18^. Much attention of late has been focused, understandably, on pesticides that target arthropods, including insecticides and acaricides that contaminate hives. Neonicotinoids in particular have been shown to have a range of adverse effects on bees even at sublethal levels; paradoxically, Kessler *et al*.^3^ demonstrated that honey bee foragers display a preference for sucrose solutions laced with neonicotinoid pesticides, absent any electrophysiological evidence that they can taste these compounds.

For their part, herbicides and fungicides have been comparatively understudied relative to the frequency with which they are documented as hive contaminants. Chlorothalonil is among the most frequently encountered contaminant in beehives, especially in wax and in pollen, where it has been found at levels up to 99 ppm^18^. The longstanding assumption has been that fungicides and herbicides, with relatively low acute toxicity relative to pesticides formulated to kill arthropods, are considered to be safe for bees. Nonetheless, fungicide and herbicides can have unexpected undesirable impacts on honey bees. The herbicide atrazine alters acetylcholinesterase activity in honey bees^19^ and exposure to glyphosate reduces sensitivity to sucrose and interferes with learning performance^20^ and navigation ability^21^. Moreover, bees consuming food contaminated with the fungicide chlorothalonil experience higher rates of infection by the parasite *Nosema* ^22,23^, reduced queen body size, fewer workers and lower colony biomass^16^. Chlorothalonil also synergizes tau-fluvalinate, a pyrethroid acaricide used in beehives for varroa control, and increases its toxicity to honey bees^24^. Moreover, the phenomenon of “entombed pollen”, whereby bees seal off cells containing pollen with higher levels of fungicide, suggests that bees may by some means recognize the presence of fungicides in their hive^25^. Although foragers bring fungicide-contaminated pollen into the hive, entombment suggests that nurse bees or other hive workers evaluate the pollen once it is in the hive and make the decision to cap off contaminated cells.

Complicating the assessment of how honey bees evaluate food quality with respect to its xenobiotic content is the fact that many of the behavioral studies to date have involved immobilization and/or force-feeding in no-choice assays. In laboratory tests, restrained bees can be induced to ingest toxic substances (*e.g.*, quinine, salicin, amygdalin and L-canavanine)^2,26^ and experience post-ingestion malaise or even death as a result^27^; bees presented with no alternative food choices will consume foods that, under choice conditions, were rejected^28^. In contrast, free-flying and freely-moving bees generally appear to detect and avoid toxic substances readily^2,29–31^. Moreover, forager responses to resources vary according to colony-level demand^32^. When foragers return from the field, they unload the nectar from their crop to receiver (or food storage) bees, which, by taking up the nectar at different rates, signal to foragers that certain food resources are preferred^33^. Thus, forager behavioral responses and decisions reflect not only an individual’s assessment of foraging resources but also a forager’s assessment of colony-level needs. Consequently, to understand forager behavioral responses to xenobiotics in natural situations, a free-flight assay of foragers that interact with hivemates is most likely to reflect natural behavior.

Accordingly, to characterize forager behavioral responses to xenobiotics when alternate food is available, we assessed their discriminatory behavior in free-flight assays in a semi-field setting. In these assays, free-flying bees from a functioning colony with nestmates present were allowed to choose between two identical feeders, one containing a test chemical in sugar water and the other containing sugar water and solvent as the control. This assay was used to compare honey bee foraging responses to natural phytochemicals and synthetic xenobiotics found as common contaminants in U.S. beehives.

## Results

Of the phytochemicals tested, at least one representative from each chemical class, albeit at varying concentrations, elicited a response indicative of either preference or avoidance (Table 1). Colony identity may have contributed to some of the variation in responses (data not shown). Caffeine, an alkaloid, was avoided by foragers according to both visitation frequency ratio at 1 ppm (one-sample *t*_(6)_ = −2.568, *p* = 0.042) and consumption ratio at 0.1 ppm (one-sample *t*_(8)_ = −4.603, *p* = 0.002). With respect to phenolic acids, evidence of discriminative behaviour was found only for sugar water containing caffeic acid; foragers showed an avoidance response according to the visitation frequency ratio at 1 ppm (one-sample *t*_(4) _= −2.908, *p* = 0.044) but showed a preference according to the consumption ratio at the same concentration (one-sample *t*_(4)_ = 23.522, *p* < 0.001).

**Table 1:** Foraging preference of foragers for natural phytochemical xenobiotics.

Category	Chemical name	Concentration	*df*	Visitation frequency ratio^1^	Sugar water consumption ratio^1^
				*mean*± *SE*	*mean* ± *SE*
Alkaloid	Caffeine	0.1 ppm	8	0.99 ± 0.04	0.93 ± 0.02**
		1 ppm	6	0.96 ± 0.02*	0.97 ± 0.02
		10 ppm	8	0.98 ± 0.05	0.98 ± 0.03
Phenolic acid	Caffeic acid	0.1 ppm	5	0.97 ± 0.04	0.96 ± 0.02
		1 ppm	4	0.91 ± 0.03*	1.08 ± 0.00***
		10 ppm	5	0.98 ± 0.04	1.04 ± 0.03
	Cinnamic acid	5 ppb	4	1.22 ± 0.14	1.11 ± 0.09
		50 ppb	1	1.11 ± 0.08	1.21 ± 0.09
		[50 ppb]^2^	[2]^2^	[1.11 ± 0.05]^2^	
		5000 ppb	2	1.08 ± 0.09	0.85 ± 0.12
	*p*-Coumaric acid	1 ppm	6	0.95 ± 0.02	0.96 ± 0.03
		10 ppm	7	0.97 ± 0.02	1.03 ± 0.03
		100 ppm	7	0.97 ± 0.03	1.00 ± 0.02
Flavonoid	Chrysin	0.1 ppm	5	0.80 ± 0.08*	0.97 ± 0.06
		1 ppm	6	1.10 ± 0.09	1.01 ± 0.06
		10 ppm	11	1.02 ± 0.04	1.06 ± 0.03
	Galangin	0.1 ppm	5	0.95 ± 0.09	1.08 ± 0.05
		1 ppm	5	1.08 ± 0.04	1.12 ± 0.05
		10 ppm	5	1.00 ± 0.05	1.00 ± 0.02
		100 ppm	5	1.11 ± 0.05	0.95 ± 0.02
	Naringenin	0.1 ppm	8	1.05 ± 0.15	1.08 ± 0.10
		1 ppm	11	0.92 ± 0.05	1.01 ± 0.04
		10 ppm	11	1.01 ± 0.07	1.00 ± 0.03
		100 ppm	5	1.00 ± 0.10	1.15 ± 0.04*
	Pinocembrin	10 ppb	7	1.01 ± 0.13	0.98 ± 0.04
		100 ppb	5	0.92 ± 0.09	1.00 ± 0.03
		1000 ppb	7	0.82 ± 0.05**	1.04 ± 0.05
	Quercetin	0.01 mM	7	1.06 ± 0.02*	1.04 ± 0.02*
		0.05 mM	7	1.24 ± 0.03***	1.17 ± 0.05**
		0.10 mM	6	1.20 ± 0.08*	1.35 ± 0.08**
		0.25 mM	5	1.26 ± 0.09*	1.37 ± 0.09*
		0.50 mM	5	1.18 ± 0.03**	1.17 ± 0.04**

^1^A ratio higher than 1 indicates a preference for the test chemical, and a ratio lower than 1 indicates avoidance of the test chemical. The asterisks indicate the means are significantly different from 1 (*p < 0.05; **p < 0.01; ***p < 0.001, one-sample *t*-test). ^2^Missing one sugar water consumption data point.

Among the five tested flavonoids, bees displayed a consistent preference response to quercetin at all five concentrations according to both visitation frequency (0.01 mM, one-sample *t*_(7)_ = 3.162, *p* = 0.016; 0.05 mM, one-sample *t*_(7)_ = 7.146, *p* < 0.001; 0.1 mM, one-sample *t*_(6)_ = 2.586, *p* = 0.041; 0.25 mM, one-sample *t*_(5)_ = 2.961, *p* = 0.032; 0.5 mM, one-sample *t*_(5)_ = 5.396, *p* = 0.003) and consumption ratios (0.01 mM, one-sample *t*_(7)_ = 2.825, *p* = 0.026; 0.05 mM, one-sample *t*_(7)_ = 3.749, *p* = 0.007; 0.1 mM, one-sample *t*_(6)_ = 4.424, *p* = 0.004; 0.25 mM, one-sample *t*_(5)_ = 3.969, *p* = 0.011; 0.5 mM, one-sample *t*_(5)_ = 4.599, *p* = 0.006). In 0.1 mM and 0.25 mM quercetin trials, foragers collected 35% more sugar water from the quercetin feeder than from the control feeder. Naringenin at 100 ppm also triggered a similar preference response (one-sample *t*_(5)_ = 3.955, *p* = 0.011); foragers collected 15% more sugar water in the case of naringenin compared with the control feeder, but the visitation frequency ratio at this concentration did not indicate a preference response (one-sample *t*_(5)_ = −0.021, *p* = 0.984). With respect to chrysin and pinocembin, bees displayed an avoidance response to 0.1 ppm chrysin (one-sample *t*_(5)_ = −2.676, *p* = 0.044) and 1 ppm pinocembrin (one-sample *t*_(7)_ = −3.539, *p* = 0.009) according to the visitation frequency ratios but neither avoidance nor preference was detected according to consumption ratio (0.1 ppm chrysin, one-sample *t*_(5)_ = −0.419, *p* = 0.693; 1 ppm pinocembrin, one-sample *t*_(7)_ = 0.833, *p* = 0.432).

### Synthetic xenobiotics

**Figure 1:**
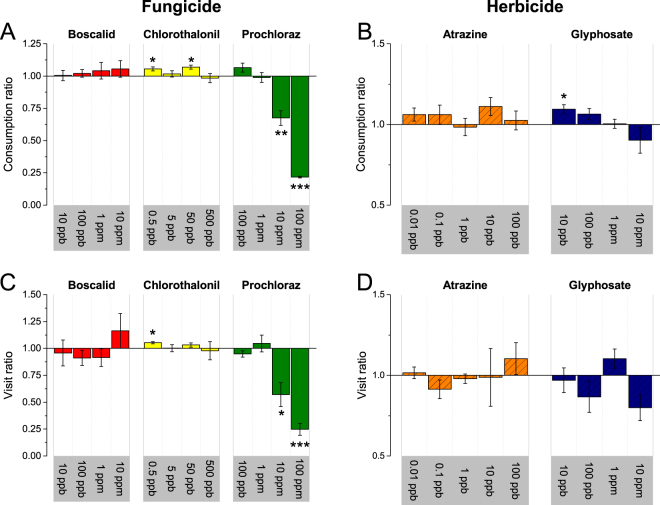
Ratios (mean ± SE) as preference indices of forager responses to selected synthetic xenobiotics, fungicides and herbicides. (**A**) Consumption ratios for three fungicides-sugar water solutions in different concentrations. (**B**) Consumption ratios for two herbicide-sugar solutions in different concentrations. (**C**) Visitation frequency ratios for three fungicide-sugar water solutions in different concentrations. (**D**) Visitation frequency ratios for two herbicide-sugar water solutions in different concentrations. A ratio higher than 1 indicates a preference for the test chemical, and a ratio lower than 1 indicates avoidance of the test chemical. The asterisks indicate the means are significantly different from 1 (*p < 0.05; **p < 0.01; ***p < 0.001, one-sample *t*-test).

Results of the free-flight preference tests with atrazine and glyphosate (herbicides) are shown in Fig. 1B,D. Foragers did not show significantly different responses to the atrazine sugar water solutions according to either consumption ratios or visitation frequency ratios. As for glyphosate, foragers displayed a preference according to consumption ratio for 10 ppb glyphosate-sugar water compared with control sugar water (one-sample *t*_(5)_ = 3.289, *p* = 0.022). At higher glyphosate concentrations, no differences in consumption ratios were detected. No difference in visitation frequency ratios was recorded at any of the tested concentrations.

Results of the free-flight preference tests with boscalid, chlorothalonil, and prochloraz (fungicides) are shown in Fig. 1A,C. Foragers showed strong avoidance responses only to high prochloraz concentrations, *i.e.*, 10 ppm (visitation frequency ratio, one-sample *t*_(5)_ = −3.88, *p* = 0.012; consumption ratio, one-sample *t*_(5)_ = −5.801, *p* = 0.002) and 100 ppm (visitation frequency ratio, one-sample *t*_(5)_ = −13.616, *p* < 0.001; consumption ratio, one-sample *t*_(5)_ = −108.626, *p* < 0.001). A preference for chlorothalonil was detected at 0.5 ppb, as indicated by both consumption ratios (one-sample *t*_(4)_ = 3.504, *p* = 0.025) and visitation frequency ratios (one-sample *t*_(4)_ = 4.781, *p* = 0.009). A similar preference for chlorothalonil at 50 ppb was evidenced by the consumption ratios (one-sample *t*_(4)_ = 4.316, *p* = 0.012) but not by the visitation frequency ratios (one-sample *t*_(4)_ = 1.588, *p* = 0.188).

## Discussion

Among all tested natural xenobiotics, foragers consistently showed a preference for quercetin according to both visitation frequency ratios and preference ratios at all concentrations. This clear predilection for quercetin under the conditions of the free-flight assay is indicative of its biological significance to honey bees. Quercetin is among the most predictable constituents of nectar, honey, pollen, beebread, and propolis. Along with kaempferol, also a flavonol, quercetin acts as a signaling substance in stimulating pollen germination and pollen tube growth^34^ and, with its derivatives, is a ubiquitous constituent of propolis in North America^35^. Beyond its value as a recognition cue indicative of appropriate food, quercetin has demonstrable health benefits for bees; among these, it up-regulates detoxification and immunity genes in honey bees^12,36^. How quercetin is detected by honey bees is uncertain; as it is non-volatile^37,38^, it may be detectable by gustatory receptors. Some nectar phenolics can modulate gustatory responsiveness in the Asian honey bee *A. cerana* ^6,39^ and may function similarly in *A. mellifera* as well.

In terms of the other phenolic acids and flavonoids tested, *p*-coumaric acid elicited neither preference or avoidance behavior at any concentration, whereas foragers displayed a preference for naringenin at 100 ppm as indicated by consumption rates and an avoidance response to both 0.1 chrysin and 1 ppm pinocembrin as indicated by visitation frequency. All of the phytochemicals tested here for their behavioral effects were examined by Mao *et al*.^12^ using qRT-PCR for their ability to upregulate CYP9Q3, the honey bee P450 with the broadest known xenobiotic substrate capacity. In their study, p-coumaric acid was the only one that elicited more than a 1.5-fold increase in expression relative to control^12^. Clearly, the ability to upregulate a key xenobiotic-metabolizing P450 gene is not correlated with differential behavioral responses of foragers to these phytochemicals.

Forager responses to caffeine appear to be complex. Honey bee foraging and recruitment to sugar water feeders containing caffeine are stimulated at the concentrations at 25 and 100 ppm^8,40^. Due to the possible pharmacological effects of caffeine on honey bee neurons^41^, the neuroactive effects of caffeine may be responsible for increasing foraging and recruitment, possibly for the benefit of the plant and to the detriment of the bee^40^. In this study, honey bees avoided caffeine at low environmental concentrations (0.1 and 1 ppm) consistent with the report by Singaravelan *et al*.^8^ that caffeine is repellent to honey bee at high concentrations (150 and 200 ppm). An individual assay also demonstrated honey bee are more likely to reject sugar water augmented with caffeine^41^. These findings indicate honey bees can detect and avoid caffeine in their food, despite its potential beneficial effects in enhancing memory^3^.

Sugar water contaminated with synthetic xenobiotics may have a discernible taste to bees. Foragers significantly avoided intake of prochloraz-sugar water at 10 ppm and 100 ppm, as evidenced by both visitation frequency ratios and consumption ratios. Nevertheless, our assays also show a significant preference for sugar water contaminated with certain fungicides and herbicides at least at some concentrations. The preference detected, however, although statistically significant, is not overwhelming, representing a difference of 1–5% between a treated feeder and a control feeder. It may be that only a subset of foragers can detect and respond behaviorally to these compounds; how they are detected, however, remains to be determined. De Brito Sanchez *et al*.^42^ have shown that taste perception of honey bees is more complex than assumed from the relatively low number of gustatory receptors. They suggest that there exist post-ingestive mechanisms in honey bees that might be as important as simple reflexive responses to chemicals; such mechanisms may have been operative in our assays.

The eusocial nature of the honey bee, however, raises a question as to which workers may experience post-ingestive malaise; whether discrimination is exercised at the flower or at the point of trophallactic contact between a returning forager and receiver bees, who then store the nectar in cells, is an open question. Honey bee colonies are known to use a complex system to signal and provide feedback to regulate foragers^43^. During trophallactic interactions between a forager and receiver bee, receiver bees learn about the nectar quality, *e.g.*, the sugar concentration, and the odor of a food source^44–46^. A forager may collect contaminated sugar water and return to the hive, delivering it to receiver bees, which may then ingest the compounds and experience post-ingestive malaise or well-being. These receiver bees, as well as the forager itself, have some capacity to signal to foragers that certain food resources should be avoided or collected by the rate at which food is unloaded^33^. Our experiments were not designed to detect social feedback, but other studies suggest that this mechanism may function in guiding forager behavior; foragers, for example, can remedy colony nutritional deficiencies by searching for complementary protein sources^47^.

If honey bees can perceive the presence of xenobiotics by gustation or any other means, another explanation of xenobiotic preference may be novelty-seeking behavior, which has been well-documented in both food scouts and nest scouts^48^. Such novelty-seeking behavior allows discovery of new resources that can enhance colony fitness. A reward system in the brain of food scout foragers could act to insure a steady supply of adequate nutrition as floral community composition changes.

Irrespective of whether food chemicals are natural or synthetic, honey bees show concentration-dependent choice patterns. Bees may well avoid a chemical in high concentrations that is preferred or ignored when present in low concentrations, such as prochloraz and naringenin, respectively. Singaravelan *et al*.^8^ found that relatively low concentrations of nicotine (2.5 ppm in 2.5–20 ppm assay and 0.5, 1 ppm in 0.5–5 ppm assay) elicited a significant feeding preference in honey bees. Köhler *et al*.^49^ observed similar preferences for nicotine at low concentrations and repellency at high concentrations. They also demonstrated behavioral response thresholds to nicotine may vary with sugar water concentrations.

Preferences for synthetic xenobiotics that are potentially detrimental can become problematical for honey bees when they are used as managed pollinators, particularly in orchard systems, where fungicides are often applied during the blooming season to prevent fungal diseases. In order to protect pollinators, fungicides are typically applied at night, with the assumption that the overnight interval is sufficient for avoiding adverse outcomes. However, in addition to the risk of direct exposure, this study suggests that the concentration of residues that persist through the next day would in fact potentially make contaminated floral resources more attractive to foragers, thereby increasing the quantity of pesticide brought back to hives. The preference for chlorothalonil on the part of the foragers demonstrated in this study, *e.g.*, may well explain its high frequency and abundance as a contaminant in beehives^18^. Moreover, some fungicides and herbicides interact not only with other agrochemicals^50^ but also with phytochemicals; although there is abundant evidence that toxicity can be enhanced by combinations of xenobiotics^51^, how these combinations affect foraging decisions has yet to be assessed, despite the implications for colony health.

## Methods and Materials

### Experimental animals

Experiments were performed with *A. mellifera*, the western honey bee. Colonies used in assays were from several satellite apiaries maintained by the University of Illinois Bee Research Facility located northeast of the UIUC campus in Urbana, IL. Colonies were relocated to the free-flight cage before use in the assay.

Bees were subjected to an acute toxicity pretest in order to determine optimal concentrations for free-flight preference assays. For these pretests, bees were collected from two hives in the same apiary. Individuals were collected at the colony entrance as they returned from foraging; five to seven foragers were placed in a small cage (12.7 cm × 5.1 cm) after collection and kept in the same cage for the assay to reduce handling stress. As a means of further reducing stress, cages were kept in the dark.

Standard five-frame colonies (containing *ca.* 4,000 worker bees with a naturally mated queen) were used for the free-flight preference assay in September-October, 2013 and June-August, 2014 at the University of Illinois Pollinatarium, located on the UIUC campus. Tested colonies were provided with a dish of ground bee pollen (Betterbee, Greenwich, NY) and a water feeder in front of their hives for the duration of the experiment. A hive inspection was carried out every two weeks to insure that the colony remained healthy and functioning normally. The colonies were replaced approximately every four weeks, when foraging activity began to decline.

### Chemicals

Two herbicides, atrazine (45330, Sigma-Aldrich) and glyphosate (45521, Sigma-Aldrich); and three fungicides, boscalid (33875, Sigma-Aldrich), chlorothalonil (36791, Sigma-Aldrich), and prochloraz (45631, Sigma-Aldrich), were obtained from Sigma-Aldrich (Milwaukee, WI). Caffeine (C0750, Sigma-Aldrich) and three phenolic acids, caffeic acid (C0625, Sigma-Aldrich), cinnamic acid (C6004, Sigma-Aldrich), and *p-*coumaric acid (C9008, Sigma-Aldrich), as well as four flavonoids, chrysin (C80105, Sigma-Aldrich), naringenin (N5893, Sigma-Aldrich), pinocembrin (P5239, Sigma-Aldrich) and quercetin (Q4951, Sigma-Aldrich), were also purchased from Sigma-Aldrich (Milwaukee, WI). One flavonoid, galangin (50-908- 908, Indofine Chemical Company, Inc.), was obtained from Indofine Chemical Company (Hillsborough, NJ).

These five synthetic xenobiotics and nine natural xenobiotics were selected for testing because they are common contaminants or constituents, respectively, of honey, pollen and propolis in U.S. hives^18,52^. The specific phytochemicals were selected because they are known to up-regulate detoxification genes^12^.

### Free-flight preference assay

The acute toxicity of each chemical-containing sugar water diet at each concentration was tested in small indoor cages (12.7 cm × 5.1 cm, modified from 2820D, BioQuip Products Inc.) before carrying out free-flight preference assays in the outdoor flight cage. This pre-test was conducted to ensure that the concentrations of the chemicals in our test did not cause acute toxicity. Foragers from a colony with a sister queen of the tested colonies were collected at the hive entrance when they returned from their foraging trip; five to seven foragers were collected and placed into a small cage, which was also used for running the tests for 48 hours. Tests of each concentration of each chemical were replicated five times. Only concentrations causing no significant difference in mortality compared with the control group and promoting at least 80% survival after 48 hours (*e.g.*, Xavier *et al*.^53^) were considered as having no actual toxicity on bees and were used in the free-flight preference assay.

In the free-flight preference assay, a large outdoor flight cage measuring 6 m × 20 m × 3 m was divided in half to yield two flight cages measuring 3 m × 20 m × 3 m. A standard five-frame colony was placed at the center of each flight cage. Artificial feeders with unscented 25% sugar water (w/v) were set up in two end corners of the flight cage equidistant from the hive (10 m). The artificial feeders had a feeder dish (14.75 cm with 24 one-mm-deep grooves that radiated from the center which allowed the bees to collect sugar water from the edge of the feeder), a 5 fl. oz. (147.87 ml) feeder cup (FC5-00090, 5.8 cm height, 7.1 cm width, Solo Cup Operating Corporation), and a feeder cup cover. The feeder cup cover was the same size as the feeder cup and had an inner foil and an opaque gray outer layer made of tape. The foil was used to prevent chemical breakdown due to exposure to sunlight; the outer tape layer insured that the feeders appeared identical to the bees so as to prevent color cues from the different sugar water from influencing the bees’ behavior.

Initially, the foragers were trained to the feeders for one or two days, after which the assays began. A trial was conducted as follows: first, 30 to 60 minutes with 25% sugar water feeders followed by 60 minutes with a 25% sugar water feeder with solvent (0.25% DMSO) vs. a treatment feeder containing 25% sugar water containing a test chemical in solvent. In order to minimize microenvironment and location effects, the locations of the control and treatment feeders were switched in the second 60 minutes. The same chemical with the same concentration was tested in both halves of the flight cages, and the treatment feeders were always placed in opposite corners of the cage (southwest vs. northeast or northwest vs. southeast) to reduce microenvironment (lights or wind) effects. Every feeder containing the sugar water to be tested was weighed at the beginning and end of every experimental step to measure the consumption of sugar water. Visitation frequency at each feeder dish was recorded by a digital time-lapse camera with snapshots at one-minute intervals. Because our pretest showed that foragers generally take five to seven minutes to return to the feeder between two successive visiting, only the pictures recorded at 6-minute intervals were used to calculate the number of bees on the feeder dish.

Two herbicides (atrazine and glyphosate) and three fungicides (boscalid, chlorothalonil, and prochloraz) as well as one alkaloid (caffeine), three phenolic acids (caffeic acid, cinnamic acid, and *p*-coumaric acid), and five flavonoids (chrysin, galangin, naringenin, pinocembrin and quercetin) were tested. To make stock solutions, phenolic acids and flavonoids were dissolved in DMSO and caffeine was dissolved in water. Every tested sugar water diet was made fresh at the tested concentration from the chemical stock solution before a test. At least three concentrations were tested for each chemical. A naturally occurring concentration of a chemical was generally tested first. Next, a ten-fold higher concentration was tested, followed by a 100-fold higher concentration. Each chemical was tested three to 12 times at each concentration with two to four colonies (usually three replicates for each concentration in each colony and at two to three concentrations per colony). The final trial numbers varied because foraging was affected by varying weather and hive conditions. Low foraging frequency can occur during severe weather or when a hive is weak, which can bias results; accordingly, those low foraging trial data were discarded.

We chose to test effects of phytochemicals on feeding preferences in a 25% sugar water solution because this concentration represents an average value in at least some plant communities. Chalcoff *et al*.^54^, *e.g.*, reported the mean nectar concentration in 26 species in a South American temperate forest species as 29.9%, ranging from 12% to 52%).

The amount of sugar water consumed from each chemical treatment feeder in two hours (one trial period) was divided by the amount of sugar water consumed from its paired control feeder to calculate the ratio as an index of preference. The sum of the number visiting each chemical’s treatment feeder in two hours was also divided by the sum of the number visiting its paired control feeder to calculate the ratio of visitation frequency. If the chemical treatment feeder and its paired control feeder were equally attractive to foragers, the ratio of sugar water consumption and the visitation frequency ratio should be equal to 1. A ratio higher than 1 indicates a preference for the test chemical, and a ratio lower than 1 indicates avoidance of the test chemical. Both the ratio of sugar water consumption and the ratio of visitation frequency were tested for normality and the mean values were tested by the one-sample *t*-test using OriginPro software (ver. 9.0, OriginLab Corporation) to test if the mean of the ratio was equal to 1.

### Data availability

The datasets generated during this study are available from the corresponding author on reasonable request.
